# Perceptions of risk from nanotechnologies and trust in stakeholders: a cross sectional study of public, academic, government and business attitudes

**DOI:** 10.1186/s12889-015-1795-1

**Published:** 2015-04-26

**Authors:** Adam Capon, James Gillespie, Margaret Rolfe, Wayne Smith

**Affiliations:** Menzies Centre for Health Policy, School of Public Health, University of Sydney, Sydney, Australia; Environmental Health Branch, Health Protection NSW, 73 Miller St, North Sydney, Sydney, Australia; University Centre for Rural Health, School of Public Health, University of Sydney, Sydney, Australia

**Keywords:** Nanotechnologies, Risk, Trust, Perception, Stakeholders

## Abstract

**Background:**

Policy makers and regulators are constantly required to make decisions despite the existence of substantial uncertainty regarding the outcomes of their proposed decisions. Understanding stakeholder views is an essential part of addressing this uncertainty, which provides insight into the possible social reactions and tolerance of unpredictable risks. In the field of nanotechnology, large uncertainties exist regarding the real and perceived risks this technology may have on society. Better evidence is needed to confront this issue.

**Methods:**

We undertook a computer assisted telephone interviewing (CATI) survey of the Australian public and a parallel survey of those involved in nanotechnology from the academic, business and government sectors. Analysis included comparisons of proportions and logistic regression techniques. We explored perceptions of nanotechnology risks both to health and in a range of products. We examined views on four trust actors.

**Results:**

The general public’s perception of risk was significantly higher than that expressed by other stakeholders. The public bestows less trust in certain trust actors than do academics or government officers, giving its greatest trust to scientists. Higher levels of public trust were generally associated with lower perceptions of risk. Nanotechnology in food and cosmetics/sunscreens were considered riskier applications irrespective of stakeholder, while familiarity with nanotechnology was associated with a reduced risk perception.

**Conclusions:**

Policy makers should consider the disparities in risk and trust perceptions between the public and influential stakeholders, placing greater emphasis on risk communication and the uncertainties of risk assessment in these areas of higher concern. Scientists being the highest trusted group are well placed to communicate the risks of nanotechnologies to the public.

**Electronic supplementary material:**

The online version of this article (doi:10.1186/s12889-015-1795-1) contains supplementary material, which is available to authorized users.

## Background

Policy makers and regulators are constantly required to make decisions despite the existence of substantial uncertainty regarding the outcomes of their proposed decisions. Over the past two decades the engagement of the public has been increasingly encouraged as a way to improve these decision outcomes in areas of environmental risk such as air pollution [1], genetically modified organisms [2], climate change [3] and nanotechnology [4-11].

Nanotechnology is a collective term for a range of technologies, techniques and processes that involve the manipulation of matter at the nanoscale – a size range from approximately 1 nanometre to 100 nanometres [12]. While consumer products developed with nanotechnology have the potential to revolutionise our way of life with new medicines, building materials and technological devices, nanoparticles, the building blocks of the consumer products made by nanotechnology, can also display unexpected and unusual forms of toxicity. Materials that are not harmful in their larger form have the potential to be toxic in their nanoform [13]. This is seen with silver which is benign in its bulk form yet is used as an antibacterial in its nanoform [14]. The uncertainty around nanoparticles is enhanced by gaps in our ability to identify or determine the presence of nanoparticle toxicity or other possible adverse impacts. Currently there is a lack of appropriate or standard tests to identify or characterise many nanoparticles as well as a lack of toxicological data [13] and population exposure measurements [11,15-18]. To further complicate matters there is an increasing sophistication of nanotechnology products as four generations (passive, active, integrated nanosystems, molecular nanosystems) of products are released into the public arena [8].

This uncertainty makes an understanding of public risk perceptions important to policy makers. There is a high potential for a dissociation between the views of scientific and technologically informed communities (including high tech business) and public reactions. There are many examples in environmental risk history where a failure to understand or align with public opinion has fed a public backlash resulting in reactive policy and regulation and/or an erosion of trust in the government. These include the Genetically Modified Organism debate in Europe and the Bovine Spondylitis Encephalopathy crisis in Britain [19]. Concerns have been raised that insensitivity to public opinion could cause similar reactions to real or imagined risks associated with nanotechnology [5,20]. Regulators and policy makers need to understand how the public perceives the risks from nanotechnology and how these perceptions differ from expert opinion. Effective regulatory decision-making must incorporate an understanding of this context of radically differing and sometimes volatile risk perceptions [21].

Our study proposes to extend and develop the knowledge base regarding perceptions of risk from nanotechnology and trust by stakeholders. To do this we use a standardised questionnaire across all the stakeholders surveyed. Secondly we examine stakeholder groups beyond highly published scientists and people attending nano conferences/working in nano laboratories that had previously been surveyed to include academic, government and business stakeholders. These three groups were chosen not just for their expertise, but because they represent the interplay of stakeholders most likely to shape policy in this field. Thirdly we seek and report on views of general risk perception (to health) and for specific products (food, cosmetics and sunscreens, medicines, pesticides, tennis racquets and computers) which broadly represent Australian regulatory arms [22]. Finally we explore several trust actors (health department, scientists, journalists and politicians), all of who have the ability to shape policy.

Our study aims to test six hypotheses. First, very little targeted research has been undertaken on differing stakeholder views of risks from nanotechnology. To explore this we hypothesise that public perceptions of risks from nanotechnology will be greater than those held by ‘experts’. Second, existing studies suggest that food and health applications of nanotechnology are likely to arouse more controversy [23]. We will test the hypothesis that the public, academics, government and business respondents will all perceive a higher level of risk in nanotechnologies that penetrate or have close and prolonged contact with the body. Three, there is inconsistent evidence that increased familiarity with nanotechnology is associated with differing perceptions of nanotechnologies [24]. Our third hypothesis proposes that public self-reported familiarity with nanotechnology will be associated with a reduction in risk perception. This relationship will be found with each of the nano products in the study. Four, the public holds less trust in the government agencies with responsibility for regulating nanotechnology than that expressed by people working in nanotechnology based industries/researching nanotechnology [23]. Our fourth hypothesis tests the evidence for this proposition. We hypothesise that the trust the public vests in scientists, the health department, journalists and politicians will be less than those held by business, academic, and government respondents who have an interest in nanotechnology.

The last two hypotheses expand on hypothesis four, examining the trust of the public in greater detail. Studies have shown that the Australian public are more likely to trust scientists and scientific institutions, followed by government agencies with industry and mass media holding the least amount of trust [25,26]. In our fifth hypothesis we test the proposition that the public will have greatest trust in scientists, followed by the health department with trust in journalists and politicians below these two. Finally, public trust in business leaders [27], science and consumer protection agencies [28] and government agencies [29] have all been associated with decreased nano risk perception. Examining other stakeholders, the greater trust that people working in nanotechnology based industries or researching nanotechnology had with scientists and government agencies, the less they perceived risk from nanotechnology [23,30]. Our sixth hypothesis is that significant negative associations exist between the trust the public vest in scientists, health department, journalists and politicians and perceived risk of nanotechnology, both when this risk is considered to health and across all risk applications. Understanding this relationship between trust and risk perception is an important avenue for risk communication and education.

## Methods

### Participation details

In March 2013 we undertook a nationally representative cross-sectional household survey of adult individuals, who provided verbal consent, using computer assisted telephone interviewing (CATI) landline (response rate = 34%) and mobile phone technologies (response rate = 19%). Sampling was based on random selection from a stratified area probability sample of private dwellings and mobile phone users. All participants were recruited through random digit dialling sampling. Sample weights accounted for the probability of selection, calibrated by age and gender (but not for jurisdictional strata) to the June 2012 Australian Bureau of Statistics (ABS) Estimated Resident Population.

From May - July 2013 we undertook a similar survey of academic, business and government stakeholders using CATI landline technology. Academic and business contacts were identified if they belong to the Australian Nanotechnology Network members list or named in the 2011 Nanotechnology – Australian Capability Report 4^th^ edition. Government contacts were identified through snowballing technique instigated by one of the authors who is actively involved in the area. A total of 1732 academic, 69 business and 45 government contacts were identified. All identified business (response rate = 36%) and government contacts (response rate = 48%) were approached while a simple random sample of academic contacts (response rate = 33%) through random sorting and selection was undertaken.

### Survey design/measures

The survey was developed from previous studies [25,31-33] and cognitively tested on 10 random individuals of varying ages and gender following Australian Bureau of Statistics guidelines [34]. Final survey measures were chosen based on cognitive understanding and easing of respondent fatigue and subject to expert review. Common survey questions were repeated verbatim across all surveys and in the same order to ensure comparability of answers and order of questions within a topic were randomised to avoid ordering effects. Participants were introduced to nanotechnology and manufactured nanomaterials, presented in a neutral fashion to minimise bias. They were told “Nanotechnology is science at a very small scale and refers to a new array of devices and materials whose key parts are 10 000 times smaller than the width of a human hair. Working at this scale allows science researchers to create new materials and products”, “Manufactured nanomaterials are the minute particles produced from nanotechnology. They are found in over 1000 products on the world market today including some food containers, cosmetics and sunscreens, clothing, sporting goods and computers”. The primary six outcome variables of interest (applications) for measuring perception of risk was based on the response to the questions “Overall, in my opinion, manufactured nanomaterials are a risk (to my health) (if they are put in the food I eat)” or “Overall in my opinion, putting manufactured nanomaterials into products such as (cosmetics and sunscreens) (medicines) (pesticides) (tennis racquets and computers) is a risk.” and were categorised into agree versus disagree. Respondents were asked to consider both risks AND benefits when answering these questions.

Independent variables included basic demographics (age, gender) and ‘nano trust’ (of the health department, scientists, journalists and politicians to keep them safe from possible health effects of manufactured nanomaterials). The public survey included questions measuring ‘nano familiarity’, while the academic, business, government survey included area of speciality.

‘Nano trust’ was determined in response to the question “In general how much do you trust X to keep you safe from any possible health effects of nanomaterials? Would you say you have no trust at all, a little trust, moderate trust, a lot of trust or absolute trust?”. The answers were then collapsed into a 3 point ‘low’ (no – a little), ‘moderate’ (moderate) and ‘high’ (a lot – absolute) response.

‘Nano familiarity’ was based on a composite of three questions. “Before today, had you heard of the term ‘Nanotechnology’” (Yes/No), if yes “Have you ever talked about nanotechnology with anyone before today?”, “Have you ever searched for information about nanotechnology?” (Yes, frequently; Yes, occasionally; Yes, only once or twice; No, never). No familiarity was based on a “no” answer to the first question, moderate familiarity on a “yes” answer to all questions, while some familiarity was the composite of the remaining answers.

The final survey instrument received ethics approval through the University of Sydney Ethics Committee. (2012/1841).

### Statistics

Data were analysed using SAS Enterprise Guide 6.1 with the Proc Survey function. The Proc Survey function allows analysis to be corrected for weighting and stratified sample design for the public survey data to provide prevalence estimates. Statistical significance between all four stakeholders was determined at a p value of ≤ 0.05 and is reported as such in this paper. For analysis of public or academic data only a secondary p value of ≤ 0.01 was also considered to account for multiple comparisons. The sample size for business and government respondents were not large enough to consider the p value of ≤ 0.01.

Due to the data structure, comparisons between stakeholder prevalence estimates of risk perceptions, trust and familiarity were undertaken by comparing overlapping 95% confidence intervals. Comparisons which contained non-overlapping confidence intervals were considered significant.

Analysis of public survey data was undertaken using descriptive statistics, chi squared analysis and paired test of proportions.

Public survey data were also analysed by unadjusted and adjusted logistic regression using the SurveyLogistic procedure. Two sets of logistic regression models were developed. The first set, consisting of 6 logistic regressions, examined the relationship between the six perceptions of risk and familiarity before and after adjusting for age and gender. As such it examined the six primary variable outcomes of interest (defined above) against nano familiarity, age and gender. The second set, consisting of 24 logistic regressions, examined the relationship between the six perceptions of risk and four trust actors before and after adjusting for nano familiarity, age and gender. All variables were retained in adjusted logistic regression models as these variables were central to the purpose of the study [35]. The Wald test was used to determine statistical significance and significant effects are also indicated with odds ratio confidence intervals that do not cross the value of 1.

## Results

### Demographics/descriptive statistics

The surveys consisted of 1355 public, 301 academic, 19 government and 21 business responses. Gender representation of the weighted public survey population was comparable to the June 2012 Australian population estimates of approximately 50% male and female. Gender representation^a^ for academic and business responses was more likely to be male (≈70%) while the gender of government respondents was almost evenly balanced.

Three hundred and ninety eight public respondents (30%) were categorised as having no familiarity with nanotechnology, while 528 (39%) were categorised as having some familiarity and 422 (31%) as having moderate familiarity with nanotechnology.

Amongst the academic responses, the best represented area of research (38%) was in the field of nanomaterials. Nanocharacterisation, nanofabrication, nanobiotechnology/nanomedicine, nanoscale theory/computation, nanophotonics, and nanoelectronics/nanomagnetics represented between 15% to 4% per discipline in descending order. The least represented discipline was translational nanoresearch (2%), of which half were involved in nanotoxicology and the other either in ethical or social research on risk/public attitudes/public impact or did not provide a sub specialisation. Of the business responses the greatest percentage of business involvement was in nanomaterial manufacture, importation or research (33% – 23%). Importation of products containing nanomaterials, waste collection/processing and legal issues had little representation. The highest representation of government respondents was health and safety (37%) followed by communication/social impact (26%), business development (16%) and environment (11%).

### Risk perception

Examining the public perception of risk through chi squared analysis we found that females were more likely to consider nanotechnology as a risk for all applications (p ≤ 0.01) and the elderly were more likely to consider nanotechnology a risk to their health, or if put in cosmetics/sunscreen, pesticides and tennis racquets/computers (p ≤ 0.01). Further details can be found in the Additional file 1: Table S1 and Additional file 2: Table S2. Using comparison of confidence intervals we found that the general public’s perception of risk was significantly higher than that found in the survey of academic, government and business opinion. This relationship was consistent whether risk was considered to health or for all 5 products (Table 1).

**Table 1:** **Perceptions of risk in different nanotechnology applications across stakeholder groups**

		**Public**				**Academic**				**Government**				**Business**			
**Application**	**Risk**	**n** ^**^**^	**%**	**95% LCI**	**95% UCI**	**n**	**%**	**95% LCI**	**95% UCI**	**n**	**%**	**95% LCI**	**95% UCI**	**n**	**%**	**95% LCI**	**95% UCI**
**Health**	Agree	798	65.7	62.2	69.2	71	26.7	21.4	32.0	4	21.1	2.2	39.9	1	5.0	0.0	14.8
**Food**	Agree	1054	84.8	82.3	87.3	144	55.4	49.3	61.4	6	33.3	10.9	55.8	4	20.0	2.0	38.0
**Cosmetics/sunscreen**	Agree	913	72.2	69.0	75.4	107	39.5	33.6	45.3	8	44.4	20.8	68.1	2	10.5	0.0	24.7
**Medicines**	Agree	866	70.8	67.5	74.0	88	32.6	27.0	38.2	3	15.8	0.0	32.6	1	5.0	0.0	14.8
**Pesticides**	Agree	808	63.8	60.3	67.3	106	39.0	33.2	44.8	6	35.3	11.9	58.7	5	23.8	5.1	42.5
**Tennis racquets/computers**	Agree	510	39.6	36.1	43.0	20	6.8	3.9	9.7	2	10.5	0.0	24.7	0	0	0	0

^^^n = number of ‘agreed’ (don’t know/refused removed).

### Comparative risk between applications

For public opinion, manufactured nanomaterials put in food were considered the greatest risk. This was followed by cosmetics/sunscreens, medicines, pesticides and tennis racquets/computers. This pattern of proportionate risk ranking was not consistent across the other stakeholders. Academic responses identified food, business responses identified pesticides and government responses identified cosmetics/sunscreens as the highest risk (Table 2).

**Table 2:** **Risk ranking of nanomaterials in products with comparison to perceived health risk, by stakeholder**

**Rank**	**Public**	**n** ^**^**^	**%**	**95% LCI**	**95% UCI**	**Academic**	**n**	**%**	**95% LCI**	**95% UCI**	**Government**	**n**	**%**	**95% LCI**	**95% UCI**	**Business**	**n**	**%**	**95% LCI**	**95% UCI**
**1**	Food**	1054	84.8	82.3	87.3	Food**	144	55.4	49.3	61.4	Cosmetics*	8	44.4	20.8	68.1	Pesticides	5	23.8	5.1	42.5
**2**	Cosmetics**	913	72.2	69.0	75.4	Cosmetics**	107	39.5	33.6	45.3	Pesticides	6	35.3	11.9	58.7	Food	4	20.0	2.0	38.0
**3**	Medicines**	866	70.8	67.5	74.0	Pesticides**	106	39.0	33.2	44.8	Food	6	33.3	10.9	55.8	Cosmetics	2	10.5	0.0	24.7
**4**	**Health**	**798**	**65.7**	**62.2**	**69.2**	Medicines	88	32.6	27.0	38.2	**Health**	**4**	**21.1**	**2.2**	**39.9**	Medicines	1	5.0	0.0	14.8
**5**	Pesticides*	808	63.8	60.3	67.3	**Health**	**71**	**26.7**	**21.4**	**32.0**	Medicines	3	15.8	0.0	32.6	**Health**	**1**	**5.0**	**0.0**	**14.8**
**6**	Computers**	510	39.6	36.1	43.0	Computers**	20	6.8	3.9	9.7	Computers	2	10.5	0.0	24.7	Computers	0	0	0	0

Note: Within each stakeholder group all 5 products were ranked by order of agreement that manufactured nanomaterials were a risk if found in that particular product. Percentage of each product agreement was compared to the generic view of risk of manufactured nanomaterials - to health (bolded).

*P value < 0.05 when compared to “health” using paired test of proportions.

**P value < 0.01 when compared to “health” using paired test of proportions.

^^^n = number of ‘agreed’ (don’t know /refused removed).

Table 2 ranks the percentage of agreement that manufactured nanomaterials are a risk to health or if placed in the 5 chosen products from highest to lowest. Also within each stakeholder group the percentage of agreement that manufactured nanomaterials are a risk to health is compared to the percentage of agreement that manufactured nanomaterials are a risk if placed in each of the 5 chosen products. The public perceived the risk of manufactured nanomaterials in food, cosmetics/sunscreens, medicines as significantly higher and pesticides and tennis racquets/computers as significantly lower than the risk of manufactured nanomaterials to health. When adjusting for multiple comparisons, the significant difference remained for all products with the exception of pesticides. Significant differences for other stakeholders can be found in Table 2.

### Public familiarity and nanorisk

The results in Table 3 show a dose response effect between increased public familiarity with nanotechnology and the reduced public perceptions of the risk of manufactured nanomaterials to health and in the 5 chosen products. The health, food, medicines and tennis racquet/computer dose response functions were stronger, showing marked changes in all three categories of familiarity, than those found in the cosmetics/sunscreen and pesticides functions. Comparing Table 3 and Table 1 shows that even the public who were most familiar with nanotechnology do not attain the prevalence of reduced risk perception as those in academia, government or business, and that the difference in these prevalences are statistically significant. Increased familiarity was associated with a significant decrease in the perception of risk of manufactured nanomaterials to health and for all products. These associations remained when adjusting for age and gender and retained their dose response nature (Table 4). In Table 4 a value less than 1 represents a reduced risk perception, while a value greater than 1 represents an increased risk perception when compared to those who had no familiarity with nanotechnology.

**Table 3:** **Public risk comparisons by familiarity**

			**No familiarity**			**Some familiarity**			**Moderate familiarity**		
**Application**	**n** ^**^**^	**Risk**	**%**	**95% LCI**	**95% UCI**	**%**	**95% LCI**	**95% UCI**	**%**	**95% LCI**	**95% UCI**
**Health**	1177	Agree	80.7	75.3	86.0	63.6	57.6	69.5	54.0	47.9	60.2
**Food**	1242	Agree	92.1	88.7	95.6	83.9	79.6	88.3	78.8	73.8	83.7
**Cosmetics/sunscreen**	1234	Agree	84.3	79.8	88.8	68.6	62.9	74.3	65.1	59.3	70.9
**Medicines**	1211	Agree	85.8	81.5	90.0	67.1	61.5	72.8	60.9	54.9	66.8
**Pesticides**	1216	Agree	75.0	69.1	80.8	59.5	53.6	65.4	58.2	52.1	64.3
**Tennis racquets/computers**	1224	Agree	55.6	49.1	62.1	36.1	30.5	41.6	28.8	23.2	34.5

Note - 95% CI used to be consistent with Table 1 as government and business had a small sample size.

^^^n = number of respondents (don’t know/refused removed).

**Table 4:** **Relationship between public familiarity and risk perception (agree versus disagree) unadjusted and adjusted for age and gender**

**Application (Risk)**	**Familiarity level**	**Unadjusted Odds Ratio**	**99% LCI**	**99% UCI**	**Adjusted Odds Ratio**	**99% LCI**	**99% UCI**
**Health ‘Agree’**	No familiarity	1 (Ref)			1 (Ref)		
	Some familiarity	0.42	0.24	0.74	0.44	0.25	0.78
	Moderate familiarity	0.28	0.16	0.50	0.30	0.17	0.55
**Food ‘Agree’**	No familiarity	1 (Ref)			1 (Ref)		
	Some familiarity	0.45	0.21	0.95	0.48	0.22	1.0
	Moderate familiarity	0.32	0.15	0.66	0.36	0.16	0.79
**Cosmetics/sunscreens ‘Agree’**	No familiarity	1 (Ref)			1 (Ref)		
	Some familiarity	0.41	0.23	0.72	0.45	0.24	0.80
	Moderate familiarity	0.35	0.20	0.61	0.40	0.22	0.73
**Medicines ‘Agree’**	No familiarity	1 (Ref)			1 (Ref)		
	Some familiarity	0.34	0.20	0.60	0.35	0.20	0.63
	Moderate familiarity	0.26	0.15	0.45	0.27	0.14	0.48
**Pesticides ‘Agree’**	No familiarity	1 (Ref)			1 (Ref)		
	Some familiarity	0.49	0.29	0.83	0.52	0.31	0.92
	Moderate familiarity	0.47	0.28	0.78	0.52	0.30	0.88
**Tennis racquets/computers ‘Agree’**	No familiarity	1 (Ref)			1 (Ref)		
	Some familiarity	0.45	0.28	0.72	0.47	0.29	0.77
	Moderate familiarity	0.32	0.20	0.53	0.35	0.20	0.60

### Perceptions of trust by stakeholders

Using comparison of confidence intervals we found that the public had significantly lower opinions of trust in scientists or the health department to keep them safe from any possible effects of manufactured nanomaterials than those held by academic or government respondents (Table 5). There were few differences between stakeholders in their trust that journalists and politicians “would keep you safe from any possible effects of manufactured nanomaterials”.

**Table 5:** **Perception of trust in trust actor, by stakeholder group**

		**Public**				**Academic**				**Government**				**Business**			
**Actor**	**Trust**	**n** ^**^**^	**%**	**95% LCI**	**95% UCI**	**n**	**%**	**95% LCI**	**95% UCI**	**n**	**%**	**95% LCI**	**95% UCI**	**n**	**%**	**95% LCI**	**95% UCI**
**Health Department**	Low	398	29.1	26.0	32.2	39	13.1	9.3	17.0	2	10.5	0.0	24.7	3	15.0	0.0	31.1
	Moderate	644	46.2	42.8	49.6	124	41.8	36.1	47.4	7	36.8	14.5	59.1	9	45.0	22.6	67.4
	High	297	24.7	21.7	27.7	134	45.1	39.4	50.8	10	52.6	29.5	75.7	8	40.0	18.0	62.0
**Scientists**	Low	322	23.7	20.8	26.6	28	9.4	6.1	12.8	1	5.3	0.0	15.6	1	5.0	0.0	14.8
	Moderate	614	44.3	40.9	47.7	101	34.0	28.6	39.4	4	21.1	2.2	39.9	8	40.0	18.0	62.0
	High	400	32.0	28.8	35.2	168	56.6	50.9	62.2	14	73.7	53.3	94.0	11	55.0	32.6	77.4
**Journalists**	Low	778	56.4	53.0	59.8	176	59.9	54.2	65.5	12	66.7	44.2	89.1	7	85.0	68.9	100.0
	Moderate	484	37.9	34.6	41.2	96	32.7	27.3	38.0	4	22.2	2.4	42.0	2	10.0	0.0	23.5
	High	76	5.7	4.1	7.2	22	7.5	4.5	10.5	2	11.1	0.0	26.1	1	5.0	0.0	14.8
**Politicians**	Low	1019	74.9	71.8	77.9	191	64.7	59.3	70.2	9	47.4	24.3	70.5	18	85.7	70.4	100.0
	Moderate	288	22.5	19.6	25.5	89	30.2	24.9	35.4	6	31.6	10.1	53.1	3	14.3	0.0	29.6
	High	31	2.6	1.4	3.8	15	5.1	2.6	7.6	4	21.1	2.2	39.9	0	0.0	0.0	0.0

^^^n = number of respondents (don’t know/refused removed).

Scientists received the most trust, followed by the health department across public, academic, government and business respondents. Journalists and politicians were the least trusted (Table 5).

### Relationship between trust and risk for the public

Public perceptions of trust in the health department, scientists and politicians were significantly associated with the reported perceptions of risk of manufactured nanomaterials to health/when in each of the five products. Table 6 displays the results for 24 adjusted logistics regressions which examine the relationship between the four trust actors and six risk perceptions after adjusting for familiarity, age and gender. ‘Low’ trust is the reference category with an odds ratio < 1 representing a reduced risk perception and an odds ratio > 1 representing an increased risk perception when compared to ‘low’ trust. Where a significant relationship existed between risk and trust (after adjustment), those with the highest level of trust exhibited a significantly lower perception of risk than those with the lowest trust level. The significant relationships included public trust in scientists and all perceived risks, public trust in the health department and all perceived risks with the exception of perceived risk of manufactured nanomaterials in pesticides, and public trust in politicians and all perceived risks with the exception of perceived risk of manufactured nanomaterials in food as well as in tennis racquets/computers (Table 6).

**Table 6:** **Association between public perception of trust and risk (agree versus disagree), adjusted for familiarity, age and gender**

		**Health Department**			**Scientists**			**Journalists**			**Politicians**		
**Application (Risk)**	**Trust level**	**Adjusted Odds Ratio**	**99% LCI**	**99% UCI**	**Adjusted Odds Ratio**	**99% LCI**	**99% UCI**	**Adjusted Odds Ratio**	**99% LCI**	**99% UCI**	**Adjusted Odds Ratio**	**99% LCI**	**99% UCI**
**Health ‘Agree’**	Low	1 (Ref)**			1 (Ref)**			1 (Ref)			1 (Ref)**		
	Moderate	0.68	0.39	1.17	0.67	0.35	1.25	1.04	0.66	1.65	0.85	0.50	1.42
	High	0.36	0.20	0.67	0.27	0.14	0.53	0.56	0.24	1.34	0.16	0.04	0.59
**Food ‘Agree’**	Low	1 (Ref)**			1 (Ref)**			1 (Ref)			1 (Ref)		
	Moderate	0.71	0.36	1.40	0.80	0.34	1.85	1.20	0.67	2.13	0.83	0.44	1.57
	High	0.37	0.18	0.78	0.34	0.14	0.78	0.51	0.18	1.47	0.21	0.05	0.83
**Cosmetics/sunscreens ‘Agree’**	Low	1 (Ref)**			1 (Ref)**			1 (Ref)			1 (Ref)**		
	Moderate	0.60	0.34	1.05	0.51	0.25	1.03	1.00	0.63	1.60	0.74	0.44	1.26
	High	0.30	0.16	0.56	0.22	0.11	0.44	0.53	0.22	1.28	0.14	0.04	0.47
**Medicines ‘Agree’**	Low	1 (Ref)**			1 (Ref)**			1 (Ref)			1 (Ref)**		
	Moderate	0.74	0.44	1.26	0.59	0.31	1.14	1.08	0.68	1.70	0.87	0.51	1.48
	High	0.44	0.24	0.80	0.27	0.14	0.53	0.52	0.23	1.18	0.20	0.06	0.61
**Pesticides ‘Agree’**	Low	1 (Ref)			1 (Ref)**			1 (Ref)			1 (Ref)**		
	Moderate	0.81	0.49	1.34	0.63	0.35	1.14	1.18	0.76	1.84	1.02	0.61	1.71
	High	0.53	0.29	0.96	0.31	0.17	0.6	0.70	0.32	1.56	0.14	0.04	0.50
**Tennis racquets/computers ‘Agree’**	Low	1 (Ref)**			1 (Ref)**			1 (Ref)			1 (Ref)		
	Moderate	0.78	0.49	1.25	0.70	0.41	1.15	1.09	0.71	1.68	0.82	0.49	1.36
	High	0.33	0.18	0.60	0.28	0.16	0.50	1.05	0.47	2.34	0.28	0.07	1.11

**p < 0.01 for overall significance of trust on risk perception for each application/actor combination.

## Discussion

Very little targeted research has been undertaken on differing stakeholder views of risk perception with regard to nanotechnology. Ho et al. [30] drew from two separate studies, one looking at public opinion, the other at the views of highly published scientists in the field of nanotechnology to compare their perceptions of risks and benefits. Although both surveys were not identical, the authors found the public perceived greater risks and less benefit for nanotechnology than the scientists, and were less likely to support funding for nanotechnology. In contrast Scheufele et al. [36] drew on the same two studies and showed that the scientists expressed more concerns than the public in the areas of pollution and new health problems with regard to nanotechnology. Siegrist et al. [23] undertook a cross sectional survey using quota sampling for the public and opportunistic sampling for experts. They found that perception of risk was significantly higher amongst the public than experts, however both had similar perceptions of benefits. The Siegrist et al. study was limited by the representativeness of both public and expert respondents with the public opinion undertaken via quota sampling and expert via attendance at a conference or working in a nano-laboratory. Our study has confirmed and enhanced this previous research in the area. We confirmed our first hypothesis that the public’s risk perception of nanotechnology is significantly greater than those working in the area of nanotechnology. This increased perception of risk is consistent regardless of whether the risk of manufactured nanomaterials is considered to oneself or if placed in one of five representative products.

Some work has been undertaken on comparative risk perceptions of specific nanotechnologies, with suggestions that food and health applications are likely to be more controversial [23]. The Australian public has shown itself less positive about nanotechnology applications that affect food and cosmetics than consumer hardware such as solar panels [25]. Further evidence has been collected to suggest that those nanotechnology applications that penetrate into the body are seen as riskier [28,37,38]. Our second hypothesis proposed that the public, academics, government and business respondents will perceive nanotechnologies that penetrate or have close and prolonged contact with the body as riskier. On this assumption, food would be seen as riskiest because of its internalisation in the body and the perception that manufactured nanomaterials are an unnatural additive to foodstuffs [5,28]. Previous research has suggested that cosmetics/sunscreens and medicines are likely to be perceived as the next most risky applications [25]. We hypothesised that their risk rating is high due to their internalisation or close prolonged contact with the body, but they are seen as less risky than food because of their beneficial to health nature. Pesticides are likely to rank after cosmetics/sunscreens and medicines because although they may be seen as hazardous, exposure will be seen as infrequent. Finally computers/tennis racquets will be seen as the least risky because of their level of contact with the body, beneficial use and lack of mental association with a hazardous product.

Our study provides evidence that both the public and academics discriminate between the risks of nanomaterials when used in different applications (Table 2). This confirms part of our second hypothesis. The survey results show that the public can differentiate between the applications of nanotechnology. The mental strategies required to undertake this differentiation were explored in our second hypothesis, which suggested that all stakeholders would rank the relative risks of nanotechnology higher or lower depending on exposure to the product, its perceived “naturalness” and its association with previous human health “disasters”. Although the concept of exposure appeared strong, with all stakeholders ranking the relative risk of manufactured nanomaterials in food or cosmetics/sunscreens as high and computers/tennis racquets as low, only the public ranked the risks as we hypothesised (food the highest risk followed by cosmetics and medicines, pesticides and finally computers). The main difference lay in the ranking of pesticides of which academic, business and government responders placed greater relative importance upon than the public (Table 2). It is difficult to interpret why this is the case. One scenario is that the relative ‘real’ risk of manufactured nanomaterials in pesticides is greater than what the public think. Another possibility is that, those working in the nanotechnology area might be more sensitive to negative perceptions of pesticides compared to the other products, or work in an area where pesticides are their interest (such as pesticide testing and regulation, especially for business and government responses where respondent numbers were low).

Examining the prevalence of risk concern, more than 60% of the Australian public agree that manufactured nanomaterials in products surveyed were a risk, excepting only consumer products such as tennis racquets and computers. Therefore in terms of regulatory importance, those sectors that are sensitive to public concern include food, cosmetics/sunscreens, medicines and pesticides. In Australia, the regulators of these sectors include Food Standards Australia New Zealand, the National Industrial Chemicals Notification and Assessment Scheme, the Therapeutic Goods Administration and the Australian Pesticides and Veterinary Medicines Authority. Overlay this with the relative importance other stakeholders place on the issues, and food and cosmetics/sunscreens are areas of greatest concern, and therefore could be seen as areas most likely to create public outrage.

One limitation of these findings comes from changing public awareness of the nanotechnology debate. The survey was administered during March 2013 just as a media debate was started around the potential risks of nanomaterials in sunscreens [39]. The ‘pro’ argument was championed by the Public Health Association of Australia, Australian Cancer Council and Australian Commonwealth Scientific and Industrial Research Organisation who based their support on Australia’s very high rates of melanoma. In this view, the demonstrable protective effects of sunscreen easily overcame the (unknown) potential risks that manufactured nanomaterials in sunscreens may present [40]. The ‘anti’ argument was championed by the environmental activist group Friends of the Earth Australia, who demanded a broader public ‘right to know’ about the use of nanomaterials. They relied on a strong version of the precautionary principle to argue that regulatory approval should be withheld until more was known about the possible detrimental effects of nanomaterials issues [41].

A review of the existing quantitative research on nanotechnology perception found inconsistent evidence on how levels of knowledge and familiarity with nanotechnology relates to perceptions [24]. The relationships between familiarity and perceptions of nanotechnology have been explored in a number of ways including surveys of support for nanotechnology in food and food packaging [29], attitudes towards nanotechnology [42], the support of use and moral acceptability of nanotechnology [43] and support for funding of nanotechnology [44]. Studies examining the interaction between nanotechnology risk perception and knowledge/familiarity have found increased knowledge/familiarity is significantly associated with a reduction in risk perception [31,45]. These results are based on views of nanotechnology as a whole. The value of this very general assessment of risk has been questioned [20], in the face of evidence that different nanotechnology applications evoke different perceptions [28,46]. These critics have advocated the assessment of nanomaterials in a more product based manner [47,48]. Our third hypothesis aimed to address these criticisms by examining the relationship between public familiarity and risk perception in a more product based manner. We found that increased public familiarity with nanotechnology was significantly associated with a reduced perception of risk. This confirms our third hypothesis. When public familiarity was compared to academic, government and business opinions using comparison of confidence intervals we found that familiarity alone could not explain the entire difference between public and academic, government and business perceptions in most cases (Tables 1 and 4). These results show that even when informed, the public is unlikely to achieve the same level of reduced risk perception in nanotechnology as those who have a professional interest in nanotechnology, a disparity important to consider in policy making. Further, it would be premature to assume from these results that increasing public familiarity with nanotechnology for example, through education, is likely to reduce perceived risks and the gap between public and academic, business and government perceptions. While familiarity was associated with a reduction in risk perception, we cannot rule out the influence cultural factors may have on these results. It should be noted that some believe values [20,45] are more important factors to consider. Another important factor to consider is message framing with qualitative studies showing, post discussion, an increase [49] or decrease [38,50] in perceived risk depending on how the message was framed. However, it can also not be ruled out that this disparity is in part induced by measurement bias, that being the measure of moderate familiarity is classified at a lower knowledge base than those who have a professional interest in nanotechnology.

Trust has long been promoted as an important factor associated with risk perception [51]. Trust reduces the complexity of decision making (or opinion formation) by accepting the risk of relying on the judgement of another party [52]. Models of trust include the causal chain mode of trust, which advocates that the direction of the association between trust and risk perception is from trust to risk perception, i.e. levels of trust shape perceptions of risk [52,53]. Within the broad trust concept, it has been argued that pre-existing knowledge, type of risk and type of trust may all influence the strength of the trust – risk perception relationship [51,54].

Trust has been shown as a significant factor when it comes to nanoparticle risk perception [24] and is an important part of the framing of public policy towards the use of nanomaterials [5,10,20]. The trust the public have in government agencies regarding nanotechnology was found to be lower than that of people working in nanotechnology based industries and nanotechnology researchers [23]. Our fourth hypothesis compared perceptions of trust between the public and academic, business and government respondents. It suggested that the trust the public vests in scientists, the health department, journalists and politicians will be less than that held by business, academic, and government officials. We partially confirmed this hypothesis by finding some significant differences between public, and academic and government levels of trust. The public do have less trust in scientists and the health department to keep them safe from any potential health effects of manufactured nanomaterials than those working in nanotechnology in academia or government.

Studies have shown that the Australian public is more likely to trust scientists and scientific institutions, followed by government agencies with industry and mass media receiving the least amount of trust [25,26]. We confirmed this in our fifth hypothesis where we found the public’s overall trust in scientists and the health department is significantly greater than the trust the public place in journalists or politicians.

Public trust in business leaders [27], science and consumer protection agencies [28] and government agencies [29] have all been associated with decreased nano risk perception. We partially confirmed this view in our sixth hypothesis where we found that those in the public who have higher levels of trust in scientists, the health department and politicians to keep them safe from any potential health effects of manufactured nanomaterials were significantly more likely to perceive lower levels of risk of manufactured nanomaterials to their health and for most products. Surprisingly, the levels of trust the public have in journalists to keep them safe from any possible health effects of nanomaterials was not associated with differing perceptions of risk. This result is unexpected, given the concern expressed over the relatively large influence the media is likely to have on public opinion in this area [5,30,55]. It is possible that this misalignment is caused through measurement bias, that is the public see the role of the journalist as an alarm bell to alert them (and the government) to the problem, rather than as a quasi-regulatory function of ‘keeping them safe’.

The results on levels of trust, hypothesis 4 to hypothesis 6, should alert policy makers to the disparity between the views of the public and ‘expert’ stakeholders. If the causal chain mode of trust, where trust affects risk perception, is applied to these results [52,53], Australian scientists and health department officials are best placed to communicate to the public about the risks and benefits of nanomaterials. However policy makers should be aware that the ‘faith’ the public bestows on scientists and health department officials may not be as large as the experts believe (or hope).

Our study contained a number of strengths. Unlike other peer reviewed publications in this area it sought opinions from a number of ‘expert’ stakeholders who are most likely to shape policy in this field. We provided a neutral definition of nanotechnology and nanomaterials. Given the potential influence framing may have on the perception of this issue, we ensured neither definition suggested benefits or risks. The concept of risk was presented in a holistic context. Respondents were asked to consider both the risks and benefits when providing a level of risk, with the Likert response scale ranging from minus two (agree strongly) to plus two (disagree strongly) to try to capture this balance. This decision was made after cognitive testing revealed that if the risk question was reversed, using the word ‘benefit’ rather than ‘risk’, the respondents perceived this ‘benefit’ question as the exact opposite of the ‘risk’ question. Risk and benefit has been justified as an inverse relationship [56], and asking about risk in this manner has been undertaken elsewhere [45,46]. Finally questions within topics were randomised and all questions that were compared across stakeholders were repeated verbatim to ensure comparability.

Our study has a number of limitations. Measurement bias may have been introduced if those who responded to our study were statistically different from those who declined to participate. In an anonymous CATI survey it is not possible to contact those who refused to participate to determine this bias. Information on academic, government and business contacts in the area of nanotechnology are difficult to acquire and all reasonable attempts were undertaken to ensure these were complete. However it is possible that our sample frame for academic, business and government did not include all those involved in the academic, business and government nanotechnology area in Australia. Also, the small number of respondents for business and government provided little power to determine associations. The response rate was similar or higher than for other groups, the sample size was an artefact of the small number of people employed in these categories. Finally, given the number of comparisons undertaken in our analysis a significance value of 0.05 meant a high probability of a type 1 error (obtaining significance by chance). Where the sample size allowed (public and academic) we also considered a significance level of 0.01. While the risk of a type 1 error was likely, many of our comparisons (and conclusions) were made across a range stakeholders or applications, with statistical significance consistent across these multiple analyses, giving us greater confidence that the effect we were detecting was real.

## Conclusion

The Australian public perceives greater risks from manufactured nanomaterials and shows less trust in scientists and the health department to provide protection from possible health effects than academic, business and government stakeholders in the nanotechnology sector. Food applications and cosmetics/sunscreens loom high on the list of public concerns, although medicines and pesticides are also causes of public concern. Policy makers should be aware of these risk and trust disparities and address public sentiment by treating nanotechnology applications in the higher risk areas with greater caution. Risk communication is best placed in the hands of trusted scientists.

## Endnote

^a^Not all gender data was collected for all academic, business and government participants.

## Additional files

Additional file 1: Table S1. Perception of risk of each nanotechnology application by gender. Additional file 2: Table S2. Perception of risk of each nanotechnology application by age group.
