# Supplementary material for: Perceptions of risk from nanotechnologies and trust in stakeholders: a cross sectional study of public, academic, government and business attitudes
Source: BMC Public Health. 2015 Apr 26;15:424. doi: 10.1186/s12889-015-1795-1 (PMC4417265; doi:10.1186/s12889-015-1795-1)
Supplement: Additional file 1: Table S1. — Perception of risk of each nanotechnology application by gender. [file 12889_2015_1795_MOESM1_ESM.doc]

Additional file 1: Table S1 Perception of risk of each nanotechnology application by gender

| **Application** |  | **Male** | | **Female** | |  |  |
| --- | --- | --- | --- | --- | --- | --- | --- |
|  | **Risk** | **Weighted n^** | **%** | **Weighted n** | **%** | **Rao Scott Chi squared**  **(df = 1)** | **p value** |
| **Health** | Agree | 345.3 | 58.9 | 442.6 | 72.2 | 14.0 | <0.01 |
|  | Disagree | 240.4 | 41.1 | 170.6 | 27.8 |  |  |
| **Food** | Agree | 485.4 | 80.8 | 568.4 | 88.5 | 9.1 | <0.01 |
|  | Disagree | 115.0 | 19.2 | 73.7 | 11.5 |  |  |
| **Cosmetics / Sunscreens** | Agree | 391.5 | 64.2 | 507.0 | 79.8 | 23.1 | <0.01 |
|  | Disagree | 217.9 | 35.8 | 128.0 | 20.2 |  |  |
| **Medicine** | Agree | 394.3 | 66.1 | 469.0 | 75.3 | 7.7 | <0.01 |
|  | Disagree | 202.4 | 33.9 | 154.2 | 24.7 |  |  |
| **Pesticide** | Agree | 341.5 | 56.6 | 439.2 | 70.8 | 16.1 | <0.01 |
|  | Disagree | 262.0 | 43.4 | 181.0 | 29.2 |  |  |
| **Computer / tennis racquet** | Agree | 196.2 | 31.9 | 295.4 | 47.1 | 17.8 | <0.01 |
|  | Disagree | 418.5 | 68.1 | 331.8 | 52.9 |  |  |

^ Note: n is weighted to account for the probability of selection, calibrated by age and gender to the June 2012 Australian Estimated Resident Population
