# Supplementary material for: Perceptions of risk from nanotechnologies and trust in stakeholders: a cross sectional study of public, academic, government and business attitudes
Source: BMC Public Health. 2015 Apr 26;15:424. doi: 10.1186/s12889-015-1795-1 (PMC4417265; doi:10.1186/s12889-015-1795-1)
Supplement: Additional file 2: Table S2. — Perception of risk of each nanotechnology application by age group. [file 12889_2015_1795_MOESM2_ESM.doc]

Additional file 2: Table S2 Perception of risk of each nanotechnology application by age group

|  |  | **Applications** | | | | | | | | | | | |
| --- | --- | --- | --- | --- | --- | --- | --- | --- | --- | --- | --- | --- | --- |
|  |  | **Health** | | **Food** | | **Cosmetics / sunscreens** | | **Medicine** | | **Pesticide** | | **Computer / tennis racquet** | |
| **Age group** | **Risk** | **Weighted n^ (Wn)** | **%** | **Wn** | **%** | **Wn** | **%** | **Wn** | **%** | **Wn** | **%** | **Wn** | **%** |
| **18-24** | Agree | 88.8 | 55.3 | 136.5 | 85.3 | 116.3 | 71.1 | 118.7 | 74.2 | 88.5 | 53.4 | 54.0 | 32.6 |
|  | Disagree | 71.7 | 44.7 | 23.5 | 14.7 | 47.4 | 29.0 | 41.3 | 25.8 | 77.3 | 46.6 | 111.7 | 67.4 |
| **25-34** | Agree | 141.9 | 57.6 | 208.4 | 83.3 | 154.5 | 61.4 | 147.6 | 59.6 | 123.8 | 50.7 | 80.2 | 31.6 |
|  | Disagree | 104.6 | 42.4 | 41.7 | 16.7 | 97.3 | 38.6 | 100.1 | 40.4 | 120.6 | 49.3 | 173.3 | 68.4 |
| **35-44** | Agree | 129.8 | 66.4 | 172.4 | 84.1 | 146.1 | 72.0 | 146.5 | 74.0 | 132.0 | 66.5 | 74.8 | 37.3 |
|  | Disagree | 65.6 | 33.6 | 32.6 | 15.9 | 56.9 | 28.0 | 51.5 | 26.0 | 66.5 | 33.5 | 126.0 | 62.7 |
| **45-54** | Agree | 146.0 | 69.1 | 184.6 | 82.7 | 160.7 | 73.3 | 149.4 | 69.1 | 151.6 | 68.5 | 87.9 | 39.2 |
|  | Disagree | 65.3 | 30.9 | 38.6 | 17.3 | 58.6 | 26.7 | 66.9 | 30.9 | 69.6 | 31.5 | 136.4 | 60.8 |
| **55-64** | Agree | 131.3 | 72.3 | 161.2 | 86.3 | 145.8 | 77.5 | 137.4 | 74.0 | 126.1 | 68.7 | 85.5 | 46.4 |
|  | Disagree | 50.4 | 27.7 | 25.5 | 13.7 | 42.4 | 22.5 | 48.2 | 26.0 | 57.6 | 31.3 | 98.6 | 53.6 |
| **65-74** | Agree | 106.9 | 72.8 | 135.5 | 86.3 | 126.3 | 79.2 | 119.5 | 75.9 | 115.7 | 75.6 | 82.0 | 52.3 |
|  | Disagree | 39.9 | 27.2 | 21.6 | 13.7 | 33.1 | 20.8 | 38.0 | 24.1 | 37.4 | 24.4 | 74.8 | 47.7 |
| **75+** | Agree | 43.2 | 76.3 | 55.2 | 91.2 | 48.9 | 82.7 | 44.2 | 80.6 | 43.0 | 75.1 | 27.2 | 48.0 |
|  | Disagree | 13.4 | 23.7 | 5.3 | 8.8 | 10.2 | 17.3 | 10.6 | 19.4 | 14.2 | 24.9 | 29.5 | 52.0 |
|  |  |  |  |  |  |  |  |  |  |  |  |  |  |
| **Rao Scott Chi squared (df=6)** |  |  | 17.2 |  | 2.6 |  | 17.0 |  | 15.7 |  | 29.2 |  | 17.2 |
| **P value** |  |  | 0.01 |  | 0.85 |  | <0.01 |  | 0.02 |  | <0.01 |  | <0.01 |

^ Note: n is weighted to account for the probability of selection, calibrated by age and gender to the June 2012 Australian Estimated Resident Population
